# Calcium-Dependent Protein Kinases in Phytohormone Signaling Pathways

**DOI:** 10.3390/ijms18112436

**Published:** 2017-11-20

**Authors:** Wuwu Xu, Wenchao Huang

**Affiliations:** State Key Laboratory of Hybrid Rice, Key Laboratory for Research and Utilization of Heterosis in Indica Rice, the Ministry of Agriculture, The Yangtze River Valley Hybrid Rice Collaboration & Innovation Center, College of Life Sciences, Wuhan University, Wuhan 430072, China; xuwuwu@whu.edu.cn

**Keywords:** CDPKs, auxins, gibberellins, ethylene, jasmonates, abscisic acid, phytohormones

## Abstract

Calcium-dependent protein kinases (CPKs/CDPKs) are Ca^2+^-sensors that decode Ca^2+^ signals into specific physiological responses. Research has reported that CDPKs constitute a large multigene family in various plant species, and play diverse roles in plant growth, development, and stress responses. Although numerous CDPKs have been exhaustively studied, and many of them have been found to be involved in plant hormone biosynthesis and response mechanisms, a comprehensive overview of the manner in which CDPKs participate in phytohormone signaling pathways, regulating nearly all aspects of plant growth, has not yet been undertaken. In this article, we reviewed the structure of CDPKs and the mechanism of their subcellular localization. Some CDPKs were elucidated to influence the intracellular localization of their substrates. Since little work has been done on the interaction between CDPKs and cytokinin signaling pathways, or on newly defined phytohormones such as brassinosteroids, strigolactones and salicylic acid, this paper mainly focused on discussing the integral associations between CDPKs and five plant hormones: auxins, gibberellins, ethylene, jasmonates, and abscisic acid. A perspective on future work is provided at the end.

## 1. Introduction

Plants have evolved multiple mechanisms for responding to varying developmental and environmental stimuli. Among these complex systems, phytohormones are important factors, allowing plants to adapt to changing environments or physical processes [1,2]. Phytohormones include auxins, cytokinins, gibberellins (GAs), ethylene (ET), and abscisic acid (ABA)—the five classical hormones discovered in the mid-20th century—as well as brassinosteroids (BRs), strigolactones, jasmonates (JA), and salicylic acid (SA), which were only later defined as phytohormones [3].

In signal transduction pathways, calcium (Ca^2+^) ions have been recognized as important second messengers for the induction of versatile signaling processes in all eukaryotic organisms. Several Ca^2+^ sensors in plants decode Ca^2+^ signals, and these include the following main families: calmodulin (CaM), calcineurin B-like (CBL), calmodulin-like proteins (CML), calmodulin-dependent protein kinases (CaMKs) and calcium-dependent protein kinases (CDPKs) [4]. Of these families, CDPKs are characterized by a unique feature whereby a Ca^2+^ sensing domain and a kinase catalytic domain are combined into a single protein. When plants are exposed to stimuli such as light, low or high temperature, high salinity, drought, hormones, and even pathogens, the cellular Ca^2+^ concentration changes in distinct spatio-temporal patterns and forms specific Ca^2+^ signals [5]. Subsequently, the CDPKs are activated by directly binding the Ca^2+^ to the calmodulin-like domain to respond to these changes and regulate the downstream targets. Various physiological processes in plants are then induced, including plant growth and development, as well as abiotic and biotic stress responses [4,6,7,8,9,10].

CDPKs have been found to be numerous, and to constitute a large family in plants, green algae and unicellular protists, but not in animals [11]. At present, 34 *CDPK* genes have been identified in *Arabidopsis thaliana*, 31 in *Oryza sativa* (rice) [12,13], at least 20 in *Triticum aestivum* (wheat), 35 in *Zea mays* (maize), and 20 in *Populus trichocarpa* (poplar) [4,13]. A large number of CDPKs have been reported to be involved in phytohormone signaling pathways in order to positively or negatively regulate plant growth, development and stress response, playing crucial roles during the growth cycle of plants. In this article, we reviewed the structure of CDPKs, along with their mechanism of subcellular localization, and focused on elucidating the biological functions of CDPKs based on their role in plant hormone signaling pathways, including auxins, GAs, ET, JAs and ABA, and provide suggestions for further research on the topic.

## 2. Structure and Subcellular Localization of CDPKs

CDPKs are characterized by their structure, which consists of a variable N-terminal domain, a typical Ser/Thr protein kinase domain, a junction domain, and a calmodulin (CaM)-like domain on which EF-hand Ca^2+^-binding sites are located [14,15,16,17]. Normally, the autoinhibitor located in the junction domain is combined with the kinase catalytic site, serving as a pseudosubstrate that maintains the CDPK protein kinases in a low basal state of activity [14]. When Ca^2+^ is increased, it binds the calmodulin-like domain and the autoinhibitory pseudosubstrate is released from the kinase domain, leading to the activation of the CDPKs. Additionally, the kinase domain binds with the ATP (or GTP) phosphate donor and transfers the γ-phosphate of ATP (or GTP) to the acceptor hydroxyl residue, thereby activating the substrate [18]. As the number and sequence of the EF-hand can diverge among some CDPKs, they exhibit different Ca^2+^ affinities and dependencies, resulting in diverse Ca^2+^ signal decoding [19].

As for the N-terminal domain, studies have revealed that it not only determines the subcellular localization, but also confers interaction with target proteins. The subcellular localizations of CDPKs are found to vary from localization in a single compartment to broad distribution throughout the cell. They have been observed to appear in the plasma membrane, cytoplasm, nucleus, endoplasmic reticulum, tonoplast, mitochondria, chloroplasts, oil bodies, peroxisomes and Golgi network [20]. REPRESSION OF SHOOT GROWTH (RSG) is a transcription factor involved in GA feedback regulation. In tobacco (*Nicotiana tabacum*), mutation at R10A in the variable N-terminal domain of NtCDPK1 reduced both RSG binding and RSG phosphorylation, in spite of leaving kinase activity unchanged, and the function of NtCDPK1 in vivo was therefore suppressed. Substituting the N-terminal domain of AtCPK9 with that of NtCDPK1 conferred RSG kinase activity [21]. StCDPK5 in potato, which was predominantly localized at the plasma membrane, activated the plasma membrane NADPH oxidase StRBOHB by phosphorylating the N-terminal region directly. Mutations of key sites in the N-terminal domain of StCDPK5 eliminated the predominant plasma membrane localization and activation of StRBOHB in vivo. A domain-swapping assay showed that the recombinant proteins for which N-terminal regions were substituted, exhibited the same localization and activation as the donors. The substitution of the N-terminal region of AtCDPK5 with that of SlCDPK2, a tomato kinase, explicitly abolished the capacity for activating and phosphorylating StRBOHB in vivo, and transferred the chimeric CDPK to the trans-Golgi network, as observed for SlCDPK2. Conversely, chimeric SlCDPK2 containing the N-terminal domain of StCDPK5 was localized to the plasma membrane and could activate StRBOHB as StCDPK5 [20].

The membrane location of CDPKs was shown to be associated with N-terminal acylation. Most CDPKs possess a predicted N-myristoylation site and cysteine residues which allow additional palmitoylation in their N-terminus [22,23]. The irreversible co-translational N-myristoylation confers CDPKs a loose basal membrane association, while stable membrane anchoring is maintained by the reversible palmitoylation. Therefore, most CDPKs are membrane-localized, and the reversibility of palmitoylation may allow CDPKs to translocate from the membrane to the cytosol or nucleus. While the myristoylation or palmitoylation sites of NtCDPK2 were respectively interrupted in tobacco (*N. tabacum*), the kinase was mainly located in the supernatant, rather than in the the microsomal pellet after ultracentrifugation of the extracts, which indicated that its subcellular membrane localization had been disturbed [24]. Several CDPKs, including AtCPK2, 3, 5, 6, 9, and 13, were shown to impede N-myristoylation and subsequent palmitoylation, while their myristoylation sites were mutated to alanine, resulting in decreased membrane association [25,26,27,28]. Additionally, AtCDPK16 is strongly predicted to be localized in the chloroplast and harbors both N-myristoylation and palmitoylation. In the wild-type form, AtCPK16 appeared predominantly at the plasma membrane, which was consistent with its predicted N-acylation. Mutation of palmitoylation that can still be myristoylated was not targeted to the chloroplasts, but was more strongly targeted to the nucleus. In contrast, mutation of the N-myristoylation site, or both sites, resulted in localization in the chloroplasts, thus suggesting that myristoylation alone is adequate to inhibit chloroplast localization in CPK16 [29].

## 3. CDPK Affects the Intracellular Localization of Substrates

It is known that CDPKs can fundamentally phosphorylate substrates, whereas little is known regarding the capacity of CDPKs to regulate the intracellular localization of substrates. In tobacco, RSG is a transcriptional activator involved in GA feedback by regulating GA biosynthetic enzymes. An earlier study exhibited that the GA levels regulated the intracellular localization of RSG, which requires the binding of 14-3-3 proteins to the phosphorylated Ser-144 in RSG [30]. The 14-3-3 proteins sequester RSG in the cytoplasm in response to GAs, negatively regulating the function of RSG. The phosphorylation at Ser-114 of NtRSG is pivotal for the interaction between 14-3-3 and NtRSG. The Ser-144 site in NtRSG was thereafter demonstrated to be phosphorylated by a calcium-dependent protein kinase NtCDPK1 [30], which proved that NtCDPK1 affected the intracellular localization of RSG. Quite recently, GhDi19-1 and GhDi19-2, two proteins of the drought-induced protein 19 family from cotton, were identified to be phosphorylated at Ser-116/-114 in the N-terminus by AtCPK11 in vitro. The S/A mutations of GhDi19-1 and GhDi19-2 showed weaker or even no phosphorylation in the presence of Ca^2+^, suggesting the crucial role of the ser site for phosphorylation. Additionally, the mutation of S/A excluded them from the nucleus, and accumulated them in the cytosol, which changed their nuclear localization. All of this indicates that this phosphorylation is necessary for proper subcellular localization [31]. It is inferred that there may be another kinase resembling AtCPK11 in cotton responsible for the phosphorylation of the two proteins and the regulation of their intracellular localization. In summary, CDPKs are required for the intracellular localization of some substrates, but more evidence is required to verify this.

## 4. CDPKs Participate in Phytohormone Signaling Pathways

CDPKs have functions in various aspects of plant growth and development, such as leaf sheath growth, root development, spikelet fertility, flowering, and senescence, as well as biotic and abiotic stress response. Many of these physiological functions are always linked with plant hormone synthesis or response pathways.

### 4.1. CDPKs Regulate Homeostasis of Gibberellins

**Figure 1:**
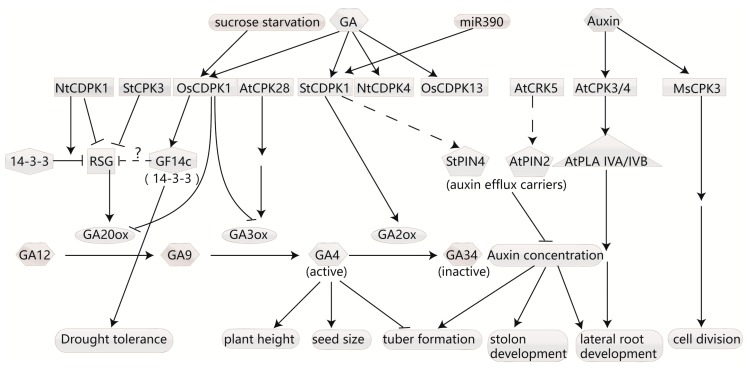
CDPKs in GA biosynthesis and auxin transport. Several CDPKs have been found to phosphorylate RSG, a basic leucine zipper (bZIP) transcriptional factor. RSG is suggested to contribute gibberellins (GA) feedback regulation by regulating transcription of genes encoding GA20ox. Additionally, the function of RSG is inhibited by binding 14-3-3 proteins to the phosphorylated sites. GA20ox and GA3ox catalyze the final steps to generate bioactive endogenous GAs while GA2ox is responsible for transferring active GAs to inactive GAs. Cellular transports of auxin are controlled by influx carriers such as the AUX/LAX influx and efflux carriers including PIN-FORMED (PIN) as well as the PGP/ABCB. Some CDPKs can phosphorylate auxin efflux carriers (PIN) to influence auxin concentration. GA and auxin affect many aspects of plants development. The dashed lines represent that there are only evidences in vitro. Question marks indicate that the relation is guessed. PLA, patatin-related phospholipase A. Arrows indicate promotion, and T bars indicate suppression.

Since a rice-membrane calcium-dependent protein kinase was found to be induced by GAs in 1995 [32], numerous CDPKs have been found to be involved in GA synthesis or signaling pathways (Figure 1). CDPKs participate in GA synthesis mainly by influencing GA20-oxidase (GA20ox) and GA3-hydroxylase (GA3ox), two enzymes that act as catalysts for the final important steps for GA biosynthesis to generate bioactive endogenous GAs [33,34,35].

In *Arabidopsis*, AtCPK28 is involved in GA homeostasis as a positive regulator. The *atcpk28* mutant showed reduced GA biosynthesis corresponding to a reduced shoot elongation phenotype when transitioning to the reproductive phase. Expression analysis exhibited that GA3ox1, catalyzing the final biosynthesis step to bioactive GA, showed a reduced expression in the *atcpk28* mutant compared with the wild-type. The growth of the mutation was consistently restored to wild-type after application of exogenous GA [36]. In contrast, the tobacco NtCDPK1 was identified as a negative modulator of GA homeostasis via the inactivation of RSG [30]. Previous studies showed that RSG is a basic leucine zipper (bZIP) transcriptional factor, and is suggested to contribute towards GA feedback regulation by regulating the transcription of genes encoding GA20ox [37]. The function of RSG is suppressed by 14-3-3 proteins [38]. GA activates NtCDPK1, resulting in phosphorylation of NtRSG. This enables 14-3-3 proteins to bind to NtRSG, and facilitates the exclusion of NtRSG from the nucleus, thereby negatively regulating GA production [30]. Additionally, gain and loss-of-function studies have found that the expression of rice OsCDPK1 is specifically activated by sucrose starvation and GA during post-germination seedling growth. Furthermore, OsCDPK1 negatively regulates the expression of GA20ox1 and GA3ox2, two enzymes essential for GA biosynthesis, and activates the expression of GF14c, a 14-3-3 protein, conferring drought tolerance in rice seedlings. Coincidently, plant height and seed size are also reduced in overexpression lines and increased in RNA interference line (RNAi-lines) of OsCDPK1 [39]. Previous studies have demonstrated that reduced GA levels promote plant tolerance to environmental stresses [40,41,42,43] including drought [44], and inhibits plant growth and development [45]. Based on this, we hypothesize that OsCDPK1 inactivates RSG proteins by up-regulating GF14c to suppress the expression of GA, leading to increased drought tolerance and decreased growth. However, direct evidence of the association between RSG and OsGF14c is still lacking.

GA2-oxidase (GA2ox) is also an enzyme involved in the GA biosynthetic pathway that transforms active GAs into inactive GAs. In potato, StCDPK1 was observed to be enhanced after 2 h of treatment with GA3 under tuberization conditions, which was accompanied by a 50% increase in GA2-oxidase expression levels and a gradually increasing expression of GA20ox [46]. It has been suggested that GAs exhibit a strong inhibitory effect, and low levels of active GAs in the stolon tip at tuber formation are essential for tuberization to proceed normally [47]. It has been conjectured that StCDPK1 triggers the degradation of GAs by increasing GA2ox content upon tuber development, and the increased GA20ox may be attributed to GA feedback regulation. Furthermore, there are some other CDPK isoforms that respond to GAs, such as NtCPK4 [48], OsCDPK13 [49], and IiCDPK2, a CDPK gene from *Isatis indigotica* [50], expressions of which were all up-regulated in response to GA treatment. However, the targets of these CDPKs and the delicate pathways regulated by GAs are still unknown.

### 4.2. CDPK Affects Auxin Transport and Responds to Auxin Signaling

The auxin indole acetic acid (IAA), which mediates cell division, enlargement and differentiation, regulates various aspects of plant growth and development from seed germination to fruit setting and growth [51]. Studies have reported that the expression and activity level of CDPKs can be induced by auxins, and adversely affect auxin concentration and response (Figure 1).

CDPKs can influence auxin level by phosphorylating their carriers. Cellular transport of auxins is controlled by influx carriers such as the AUX/LAX influx and efflux carriers, including PIN-FORMED (PIN) as well as the PGP/ABCB (for P-glycoprotein/ATP binding cassette protein subfamily B) transporters, several of which cooperate with PINs [52,53,54]. In addition to the suggestion that StCDPK1 may play a role in GA-signaling during potato tuberization, it has recently been observed to be regulated by miR390 at the posttranscriptional level and to phosphorylate the auxin efflux carrier StPIN in vitro [55]. As the isoform is an active plasma membrane-associated kinase [46,56], it is conceivable that StPIN may be a potential targets of StCDPK1 in vivo. In stolons and tubers, GUS activity driven by the StCDPK1 promoter overlaps with the high auxin concentration [57], and StCDPK1 in the vascular tissue of stolons and tubers shows a similar expression pattern as long looped PINs, StPIN4 and StPIN2 [58], revealing that this kinase may regulate auxin transport by phosphorylating StPIN. Above all, it has been inferred that StCDPK1 regulates tuberization by the crosstalk between GA synthesis and auxin transport. Further evidence has been provided for the plasma membrane-localized CDPK-related kinase 5 (AtCRK5). Atcrk5 mutants have shown reduced auxin levels in their root tips and delayed gravitropic responses, along with the phenotype exhibiting more lateral roots compared to the wild-type. Furthermore, PIN2 was observed to accelerate the accumulation in brefeldin bodies in the crk5 mutant. Kinase assays demonstrate that AtCRK5 phosphorylates AtPIN2 in vitro. The phenotypic differences in the mutants may be the result of reduced auxin levels caused by impaired AtPIN2 phosphorylation [59].

In addition to phosphorylating aux carriers, CDPKs can also participate in the auxin response pathway. *Arabidopsis* AtCPK3 and AtCPK4 have been found to phosphorylate AtPLA IVA and IVB, two patatin-related phospholipase A enzymes [60]. Plant PLA (pPLA) inhibitors have been observed to inhibit the expression of several Auxin/IAA genes and the engineered auxin-activated DR5 promoter [61]. Mutants of AtPLAIVA displayed reduced lateral root development, which is a characteristic of an impaired auxin response. AtPLAIVB is transcriptionally induced by auxin [60]. Taking all this into consideration, AtCPK3 and 4 may function on PLAs to modify the auxin signaling pathway.

Studies have reported that CDPKs can be regulated by auxins at the transcriptional and activity level. For instance, the kinase *MsCPK3* gene in *Medicago* exhibited an increased expression in response to treatment with high concentration of auxins (2,4-D) [62]. An early inhibitor study showed that the activity of a 50 kDa CDPK in cucumber was increased after 1 day of IAA treatment during adventitious root formation [63]. However, the underlying mechanism still needs to be explored.

### 4.3. CDPKs Respond to Ethylene and Affect Ethylene Biosynthesis

**Figure 2:**
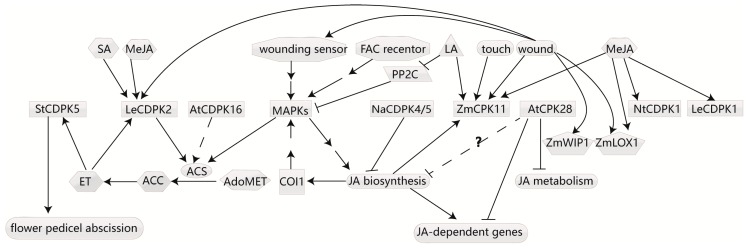
CDPKs affect ethylene and jasmonic acid biosynthesis and respond to several signals. The expression of LeCDPK2 can be aroused by ethylene (ET), methyl jasmonate (MeJA), salicylic acid (SA) and mechanical wounding. ACS, the key ethylene biosynthesis enzyme, can be phosphorylated and stabilized by LeCDPK2 and MAPKs to respond to wounding signaling. CDPKs in the wounding response show frequent crosstalk with MAPKs. Furthermore, MAPKs are required for JA biosynthesis, which is repressed by NACDPK4 and 5. AtCPK28 has been demonstrated to inhibit JA metabolism and the expression of some JA-dependent genes. It seems that AtCPK28 may also repress JA biosynthesis. JA, jasmonic acid; LA, linolenic acid. The dashed lines represent that there is only evidence in vitro. Question marks indicate that the relation is guessed. Arrows indicate promotion, and T bars indicate suppression.

Ethylene is involved in multiple processes in plant growth and development, including seed germination, cell elongation, leaf and flower senescence and abscission, fruit ripening, nodulation, and response to stimuli. CDPKs can also respond to ET signaling and affect its biosynthesis. The key ET biosynthesis enzyme 1-aminocyclopropane-1-carboxylate (ACC) synthase (ACS) is responsible for converting S-adenosylmethionine (AdoMet) to ACC [64], which is the rate-limiting step in ET biosynthesis, playing an important role in the ET biosynthesis pathway. Several CDPKs have been reported to phosphorylate ACS, resulting in changed ET content (Figure 2).

In *Arabidopsis*, the nine ACS proteins can be divided into three main types based on their C-terminal sequences [65]. Both the type I and II proteins have a conserved serine residue that can be phosphorylated by CDPKs [66,67,68], while type III proteins have only a very short C-terminal extension lacking this phosphorylation site for CDPKs [69]. Nevertheless, AtACS7, which belongs to the type III ACS and is involved in root gravitropism in a calcium-dependent manner, was found to be phosphorylated by AtCDPK16 at Ser216, Thr296, and Ser299 in vitro [68]. In tomato fruit tissue, LeCDPK2 was able to phosphorylate LeACS2 at Ser-460. LeACS2 was immediately modified by CDPK and mitogen-activated protein kinase (MAPK) at different sites at the post-translational level in response to wound signaling, and LeACS2 stability required phosphorylation at both sites [70]. However, a previous study has shown that the expression of LeCDPK2 could also be aroused by ET, methyl JA, SA, and mechanical wounding [71]. It’s assumed that LeCDPK2 not only participates in ET biosynthesis, but also responds to ET signaling. In addition, proteomics profiling of ET-induced tomato flower pedicel abscission showed that CDPK5 and SRL3 increased in response to ET [72]. It’s known that CDPKs phosphorylate ACS to regulate ET biosynthesis, but the manner in which CDPKs participate in ET signal transduction requires further investigation.

### 4.4. CDPKs Regulate JA Biosynthesis

JA is influenced by a variety of plant developmental and physiological processes, including biotic and abiotic stress tolerance, oxidative defense, reproductive processes, fertility, sex determination, storage organ formation, fruit ripening and senescence, root elongation, and interaction with other hormones [73]. Several CDPKs have been reported to regulate JA biosynthesis or be regulated by JA (Figure 2).

In tobacco, NaCDPK4 and NaCDPK5 were redundantly responsible for JA biosynthesis. While they were simultaneously silenced, IRcdpk4/5 plants accumulated exceptionally high JA levels with the stunted-growth phenotype and the abortion of most flower primordia. Genetic analysis has indicated that high JA levels increase the activity of SA-induced protein kinase, a mitogen-activated protein kinase (MAPK) in IRcdpk4/5 plants, dependent on CORONATINE INSENSITIVE1 (COI1) [74,75]. As MAPKs are required for JA biosynthesis [73,76,77], this suggests that JA signaling cross-talks with MAPK, and NaCDPK4 and 5 participate in regulating JA homeostasis. However, the direct targets of NaCDPK4 and 5 are absent and the fine regulation pathway needs further exploration.

AtCPK28 regulates GA synthesis [36], but has recently been demonstrated to spatiotemporally alter JA. The Atcpk28 mutant exhibited reduced growth and showed elevated expression of JA-dependent genes as well as high levels of several JA metabolites in a growth phase-dependent manner. The accumulation of JA metabolites in the mutant was limited locally to the central rosette tissue, whereas JA content exhibited no alteration in the early stages. Resistance of the mutant towards herbivores or necrotrophic pathogens showed no change in the generative growth phase. Coincidently, the growth phenotype of atcpk28 can be fully reversed by the abolishment of JA biosynthesis or JA signaling, but not by the modification of GA signaling [78]. Regulation of crosstalk between JA and GA is crucial for plant development by adjusting energy investment in defense mechanisms and plant growth [79,80].These suggest that AtCPK28 acts as a growth phase-dependent key negative regulator of distinct developmental processes by balancing JA and GA.

Not only are CDPKs involved in JA biosynthesis, but they also respond to JA signaling. In maize, ZmCPK11 is regulated by linolenic acid (LA) and methyl jasmonate (MeJA) at the enzymatic and transcriptional level, and is involved in wound and touch signaling. Overexpression of ZmCPK11 in *Arabidopsis* showed kinase activation upon wounding and touching. Furthermore, the wound-induced activation of ZmCPK11 in maize and the transgenic *A. thaliana* plants was eliminated by pre-treatment with acetylsalicylic acid, an inhibitor of JA-dependent wound signaling [81]. Additionally, NtCDPK1 in *N. tabacum* [82], wound responsive LeCDPK1 in tomato leaves [83], and two CDPKs in rice [84] were also induced by MeJA at the expression level. Conversely, StCDPK2 in potato was down-regulated by JA at both the expression and activity levels [85].

### 4.5. CDPKs Participate in ABA-Induced Stomata Movement and Respond to ABA Signaling

**Figure 3:**
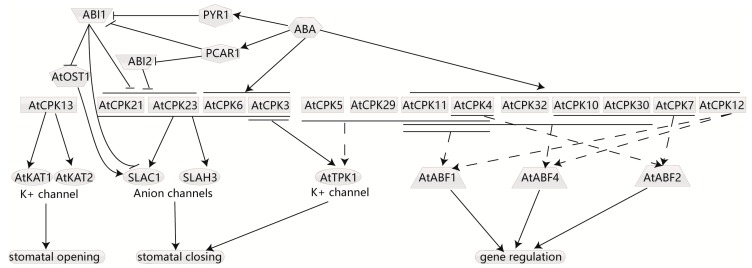
CDPKs involve ABA-induced stomata movements and phosphorylate ABFs. CDPKs participate in ABA signal transduction by affecting the activity of ABA-responsive element-binding factors/proteins (ABFs) and are involved in ABA-dependent stomata movement. Many CDPKs in Arabidopsis have been found to phosphorylate ABFs in vitro, but evidence in vivo is lacking. CDPKs phosphorylate K^+^ channel (AtTPK1) and anion channels (SLAC1 and SLAH3), triggering the release of K^+^ and anions from guard cells, resulting in stomatal closure. Some phosphorylate two inward K^+^ channels (AtKAT1 and AtKAT2) and inhibit their activity, leading to stomatal opening. PYR/PYL/RCAR–PP2Cs–SnRK2s is the core signaling pathway of ABA from ABA perception to ABA-regulated gene expression. The dashed lines represent that there is only evidence in vitro. Arrows indicate promotion, and T bars indicate suppression.

The phytohormone ABA is implicated in developmental progress and resistance to stress [86]. The core signaling pathway of ABA, PYR/PYL/RCAR–PP2Cs–SnRK2s has been established from ABA perception to ABA-regulated gene expression [87]. However Ca^2+^ signals always accompany ABA signaling transduction, initiate the regulation of ion channels, and change the expression of downstream genes, which require Ca^2+^ signal decoders, including CDPKs. Reverse genetics has proved that CDPKs are indeed connected with ABA and the stress response [88,89,90] (Figure 3). For instance, the overexpression and knockout of AtCPK4 or AtCPK11 in *Arabidopsis* enhanced and inhibited plant ABA- and salt-sensitivity with the expression of several ABA-responsive genes being altered [90].

ABA can be triggered by various stimuli. When plants suffer drought stress, ABA is induced and transported to the stomata to trigger K^+^ and anions release from the guard cells, resulting in stomatal closure [91,92]. Active ion channels are required for this process. Several CDPKs have been found to function in ion channels to regulate stomatal closure in response to ABA. In *Arabidopsis*, AtCPK3, 6, 21, and 23 as well as SnRK2 kinase open stomata 1(AtOST1) were identified to activate two anion channels, the slow-type anion-associated 1(AtSLAC1) and its homologue ATSLAH3 [89,93,94]. AtCPK3 can phosphorylate the tandem-pore K^+^ channel 1 (AtTPK1) at an N-terminal site, which is imperative for the binding of 14-3-3 proteins and efflux of K^+^ from the vacuoles [95]. Additionally, other CDPKs, such as AtCPK4, 5, 11, and 29, were also capable of phosphorylating AtTPK1 in vitro [95]. However, CDPKs also participate in stomatal opening. AtCPK13, predominantly expressed in *Arabidopsis* guard cells, has been found to phosphorylate two inward K^+^ channels, AtKAT1 and AtKAT2, and inhibit their activity [96]. But AtCPK13 may act in an ABA-independent manner, as AtCPK13-overexpressing mutants showed no default in ABA-triggered stomatal closure [96], and ABA signal transduction showed no impact on AtCPK13 expression [97].

CDPKs can also participate in ABA signal transduction by affecting the activity of ABA-responsive element-binding factors/proteins (ABFs/AREBPs). *Arabidopsis* kinase AtCPK32 was identified to interact with AtABF4 in vitro. Overexpression of AtCPK32 exhibited ABA-hypersensitive phenotypes, and the mutation of the phosphorylated sites showed decreased ABA-induced transcriptional activity of AtABF4, suggesting that phosphorylation by AtCPK32 activates the transcription factor [88]. Additionally, a yeast two-hybrid assay attested that AtCPK10 and 30 also interacted with AtABF4 [88]. Additionally, AtCPK 4, 7, 10 and 30 were found to interact with AtABF2 in vitro [26]. As AtCPK7, 10, and 30 were localized in the plasma membrane, it is uncertain whether their interaction with the transcription factor exists in vivo. Furthermore, AtCPK4, 11 and 12 were able to phosphorylate two ABA-responsive transcription factors, AtABF1 and AtABF4, in vitro [98,99]. A loss-of-function mutant of AtCPK4 and 11 showed multi-effect ABA-insensitive phenotypes in seed germination, seedling growth, and stomatal movement and decreased tolerance of seedlings to salt stress [98]. These kinases are more likely to be upstream regulators of ABFs for their partial nuclear localization.

## 5. CDPK Involved in the Cross-Talk of Multiple Signaling Pathways

CDPKs play an important role in multiple signaling pathways induced by abiotic stress, including drought, salt, cold, and heat, and by biotic stress such as fungus, as well as by plant hormones.

In rice, OsCDPK13 was found to be highly expressed in the roots and leaf sheaths of seedlings. The expression of OsCDPK13 in the leaf sheath segments was increased in response to GA [49,100,101] and induced by cold stress [100,101,102], but was suppressed in response to ABA, brassinolides, salt, and drought stresses [100]. Antisense OsCDPK13 transgenic rice lines were observed to be shorter than the control lines [100], and showed a lower accumulation of aldolase [49]. Konishi et al. showed that both the protein and transcript levels of aldolase were increased in the roots of wild rice following GA3 treatment, and promoted the root growth of rice seedlings. It was suggested that GA3 up-regulates the aldolase level via the CDPK13 in rice roots to accelerate root growth. OsCPK12 is also involved in multiple signaling pathways, including salt tolerance and blast resistance. Gain and loss-of-function studies demonstrated that OsCPK12 promotes tolerance to salt stress by controlling the expression of OsAPx2, OsAPx8 and OsrbohI to reduce the accumulation of reactive oxygen species. Overexpression of OsCPK12 exhibited increased plant sensitivity to ABA and susceptibility to blast fungus, while suppression of OsCPK12 in rice did not affect the response to ABA, and showed no significant difference in blast resistance compared with the wild-type, which suggested that OsCPK12 positively regulates the ABA signaling pathway and negatively regulates blast resistance, and that the functional redundancy among OsCPK12 and other rice CDPKs exists in the ABA signaling pathway of rice and in the blast disease resistance pathway [10]. OsCPK4 is a plasma membrane-localized protein kinase [103]. The expression of OsCPK4 is induced by high salinity, drought, and ABA. Overexpression of OsCPK4 showed enhanced tolerance to salt and drought stress via stronger water-holding capability and reduced membrane lipid peroxidation and electrolyte leakage, which protects cellular membranes from stress-induced oxidative damage [103] and enhances resistance to blast disease by preventing fungal penetration [104]. Research on CDPKs in biotic stress defense has been frequently reviewed. CDPK activation was initiated during pathogen defense [105,106,107], which resulted in various reactions, including ROS production, hypersensitive response, regulation of immune gene expression [106], as well as changes in phytohormone biosynthesis and signaling [107] to regulate diverse plant immune responses. This may contribute greatly to the exploration and application of plant disease resistance mechanisms. Importantly, the roles of CDPKs in different signaling pathways should be elucidated, and the intertwined relationship between these signaling pathways should be discussed and summarized.

## 6. Perspectives

Many CDPKs have been found to phosphorylate substrates in vitro without regard for their subcellular localization. Further evidence in vivo is required. Some assays that require the sequence to be attached to N-terminal domain of CDPKs may influence the subcellular localization of the CDPKs and further affect the interaction between the kinase and its targets. Considering this, assays regarding localization and interaction should refrain from changing the N-terminal domain of CDPKs. The mechanism for membrane anchoring has been much studied and elucidated, whereas theories for other subcellular localizations are still lacking.

CDPKs are associated nearly all phytohormones in plants. Currently, CDPKs in ABA, GA, auxin and ET signaling transduction have been studied the most—particularly in ABA—but little has been done on the cytokinin signaling pathway. The association between CDPKs and newly defined plant hormones, such as BR, JA, and SA, have not been researched in detail. As numerous CDPKs have been reported to respond to several hormones or regulate their biosynthesis simultaneously, investigating the roles of CDPKs in multiple signaling networks may provide new avenues for clarifying the crosstalk between these hormones.
